# Bilobalide Alleviated Dextran Sulfate Sodium-Induced Experimental Colitis by Inhibiting M1 Macrophage Polarization Through the NF-κB Signaling Pathway

**DOI:** 10.3389/fphar.2020.00718

**Published:** 2020-05-21

**Authors:** Heng Zhang, Nengqi Cao, Zhilong Yang, Xingchao Fang, Xinyu Yang, Hao Li, Zhi Hong, Zhenling Ji

**Affiliations:** Department of General Surgery, Nanjing Lishui District People’s Hospital, Zhongda Hospital Lishui Branch, Southeast University, Nanjing, China

**Keywords:** natural product, bilobalide, colitis, M1 macrophage polarization, NF-κB signals

## Abstract

Bilobalide, a unique *Ginkgo biloba* constituent has attracted significant interest as a novel therapeutic option for neuronal protection. However, there is paucity of data on its effect on colitis. This work sought to evaluate the effect of bilobalide on macrophage polarization *in vitro* and dextran sulfate sodium (DSS) induced colitis *in vivo*. Through the 3-(4,5-dimethylthiazol-2-yl)-2,5-diphenyltetrazolium bromide (MTT) and annexin V/PI assay, it was shown that bilobalide has no significant toxicity on macrophage. Lipopolysaccharide (LPS) and interferon-gamma (IFN-γ) induced macrophage activation and polarization were significantly suppressed by bilobalide as indicated by reduced expression of cytokine, major histocompatibility complex II (MHC-II), and CD11c. Pertinently, the signaling pathway study showed that the phosphorylation of p65 and its nuclear translocation were decreased while STAT1 was not affected. In DSS-treated mice, administration (i.g) of three doses of bilobalide na\mely 1.25 mg/kg (low dose group), 2.5 mg/kg (medium dose group), and 5 mg/kg (high dose group) was performed daily starting from day 1 to day 10. Medium and high dose bilobalide markedly reduced the inflammation of colitis proved *via* elevation of bodyweight, decrement in disease activity index (DAI), alleviation of colon damage as well as reduction in activity of colon tissue myeloperoxidase activity. In accordance with the *in vitro* results, the levels of inflammatory cytokines such as interleukin 6 (IL-6), IL-1β, and tumor necrosis factor (TNF-α) in serum as well as messenger RNA (mRNA) expression in colon were obviously reduced in the bilobalide treated groups. Also, factor nuclear factor kappa B (NF-κB) signaling pathway was decreased significantly by bilobalide treatment. Collectively, these results indicated that administration of bilobalide improved experimental colitis *via* inhibition of M1 macrophage polarization through the NF-κB signaling pathway. Thus, bilobalide could act as a potential drug for the treatment of inflammatory bowel disease (IBD) in the not-too-distant future.

## Introduction

Inflammatory bowel disease (IBD) is a multifactorial complex illness which principally consists of ulcerative colitis and Crohn’s disease. Physiologically, the human body ensures the removal of detrimental stimuli through inflammation, nevertheless, several outcomes such as tissue damage may take place with an uncontrolled inflammation response which must be tightly controlled (Medzhitov, 2008). In order to validate potential treatments in preclinical settings and decipher the pathogenesis of IBD, a series of mouse colitis models have been developed as indispensable tools. Usually, the colitis mouse model induced by dextran sulfate sodium (DSS) has been used extensively in human ulcerative colitis research due to its fast, flexible, reproducibility and controllability (Mei et al., 2019). The DSS acts as a negatively charged water-soluble sulfated polysaccharide with a highly variable molecular weight ranging from 5 to 1,400 kDa (Chassaing et al., 2014). Through the alteration in the concentration and the frequency of DSS administration, acute, chronic, and relapsing models of intestinal inflammation may be achieved (Di Martino et al., 2016; Nunes et al., 2019). Notably, a closely resembled human ulcerative colitis (UC) is regarded as the most severe murine colitis that is induced through the administration of 40 to 50 kDa DSS in drinking water (Okayasu I et al., 1990). Pathomechanistically, DSS may induce inflammation *via* activation of transcription factor nuclear factor kappa B (NF-κB) signaling pathway, which is principally localized in the colon with characteristics such as ulceration, damage in epithelial cells, hyperplasia in lymphoid cells, and inflammatory infiltration in mucosa cells.

Actually, continuous or discontinuous mucosal inflammation, ulceration of the mucosa epithelial, cell destruction, and ulceration of most intestine and/or colon (Zigron and Bronstein, 2019) characterized both UC and Crohn’s diseases. Based on existing evidence, immune responses that are elicited by inflammatory cell infiltration, especially macrophages are associated with IBD (Shi et al., 2019; Zeng et al., 2019). Macrophages are reported to be central mediators of innate immune homeostasis and inflammation, which can migrate to the inflamed colonic mucosa and release active metabolites of oxygen, nitrogen, and proteases for the degradation of the extracellular matrix (Keane et al., 2017). Different microenvironment stimulation has been reported to trigger macrophages polarization into inflammatory M1 phenotype and anti-inflammatory M2 (Kim et al., 2019; Lokhonina et al., 2019). The M1 macrophages, usually act as efficient effector cells in the response to microbial products or interferon-gamma (IFN-γ) (Abraham and Medzhitov, 2011), and is characterized by high levels of pro-inflammatory cytokines such as tumor necrosis factor (TNF-α) (Barger et al., 1995), nitric oxide (NO), and reactive oxygen species (ROS) intermediates. This process therefore promotes polarized type I immune responses by mediating host defense against bacterial, protozoal, and tumor cell infections (Haffner-Luntzer et al., 2019; Sun et al., 2019). The M2 macrophages are involved in the regression of inflammation, and are usually activated by interleukin (IL)-4 and IL-10 amidst their involvement in tissue repair and remodeling processes (Zhang et al., 2018; Yu et al., 2019). Some studies have shown that M1 macrophages increased in colitis, while M2 macrophages decreased under the same condition culminating in phenotypic imbalance of macrophages, which further promoted the development of colitis (Feng et al., 2019; Song et al., 2019). Pertinently, the switching of M1 to M2 macrophages could decrease colitis (Wang et al., 2019), indicating that the regulation of macrophages polarization could act as the emerging targets for IBDs therapy (Lin et al., 2014; Zhu et al., 2016; Zhu et al., 2018).

As a very valuable herb, *Ginkgo biloba*, has been used as a traditional herbal medicine (TCM) for the past several thousand years. *G. biloba* leaf extracts showed a variety of biological activities, mostly against cardiovascular and neurological diseases (Wu et al., 2008; Ong Lai Teik et al., 2016; Zhang et al., 2016; Al-Adwani et al., 2019). Recently, many studies have hinted that anti-inflammation activity contributed to the improvement of cardiovascular and neurological diseases by *G. biloba* leaf extracts. The *G. biloba* extract reduced hippocampus inflammatory responses and improved cardiac functions and depressive behaviors in a heart failure mouse model (Zhang et al., 2019). The *G. biloba* inhibited hydrogen peroxide induced activation of NF-κB in vascular endothelial cell (Wei et al., 1999) and inhibited cerebral ischemia/reperfusion induced inflammatory response in astrocytes *via* TLR4/NF-κB pathway in rats (Li et al., 2020). The extracts also showed high inhibition of TNF-*α*-induced vascular cell adhesion molecule-1 (VCAM-1) release, which was partly due to the NF*-*κB pathway impairment (Piazza et al., 2019).

Moreover, the anti-inflammatory activities of *G. biloba* extract was gradually confirmed. It has been reported that the phospholipase A2 could be inhibited by the extract of *G. biloba* leaves, wherein the extracted compound showed the potent antiarthritic activity in rat adjuvant-induced arthritis (Middleton, 1992). There is also evidence for an anti-inflammatory function of *G. biloba* extract, with reports that *G. biloba* inhibited formalin and carrageenan-induced paw edema and reduced experimentally induced uveitis in rats (Abdel-Salam et al., 2004; Ilieva et al., 2004). These anti-inflammatory properties of *G. biloba* are suggested to be mediated through inhibition of a range of proinflammatory mediators including NO, IL-1β and tumor necrosis factor α (TNF-α) (Tonussi and Ferreira, 1992; Kobuchi et al., 1997; Cheung et al., 1999; Wadsworth, 2001; Ilieva et al., 2004). There is a unique sesquiterpene trilactone constituent (bilobalide) in *G. biloba* which has shown anti-inflammatory properties *via* lowering production in of some inflammatory cytokines (Goldie and Dolan, 2013). However, extensive literature and patent searches indicate that there is data scarcity on the effect of bilobalide on colitis

Herein, the effect of bilobalide on macrophages polarization in murine RAW264.7 cell line was analyzed, while the effect on DSS-induced colitis was evaluated. Through *in vitro* and *in vivo* experiments, bilobalide was hypothesized to exert its anti-inflammatory activities in colitis by inhibiting macrophages M1 polarization *via* suppression of NF-κB signaling pathway.

## Materials and Method

### Animals

C57BL/6 mice (female, 6–8 weeks old, 18–22 g) were purchased from the Laboratory Animal Centre of Jiangsu University (Zhenjiang, China). The mice were grouped randomly at specific-pathogen-free (SPF) facility, while the temperature was controlled at 22 ± 2°C and the lighting conditions were constantly circulated in 12 h of light and 12 h of darkness. The animal experiments were approved by Animal Experimental Ethical Committee of Southeast University and carried out at Laboratory Animal Research Centre of Jiangsu University (NO. 2018–0553). Every effort was made to reduce the number of animals used coupled with minimizing the suffering of the animals.

### Reagents

Bilobalide (S2276, 99.09% purity) was obtained from Selleck Co. Ltd. (Shanghai, China). Bilobalide was dissolved in dimethyl sulfoxide (DMSO) at 30 mM as a stock solution for *in vitro* experiment. It was diluted with culture medium when used and the some concentration of DMSO was used as vehicle control. As for animal administration, bilobalide were dissolved in the sodium salt of carboxymethyl cellulose (CMC-Na, 0.9%). Lipopolysaccharide (LPS, L2630) was bought from Sigma-Aldrich (St. Louis, MO, USA). The DSS was provided by MP Biomedical (molecular weight 36,000–50,000, MP Biomedical ICN, France). Roswell Park Memorial Institute Medium (RPMI)-1640 and fetal bowel serum were supplied by from Life Technology (Carlsbad, CA, USA). Antibody for CD11C-pecy5 (15-0114-82), MHC-II-PE (12-0920-82) was obtained from eBioscience (San Diego, CA, USA). Recombinant murine IFN-γ (315-05) and macrophage-colony stimulating factor (M-CSF, 315-03) were provided by PeproTech (Rocky Hill, NJ). Antibodies for p-p65 (3033) and p65 (8242) were bought from Cell Signaling Technology (Beverly, MA, USA). Antibodies for p-STAT1 (9167) and STAT1 (14994) were obtained from Santa Cruz Biotechnology (Santa Cruz, CA). Alexa Fluor 488 goat anti-rabbit IgG (A-31565) and 4′, 6-diamidino-2-phenylindole (DAPI) were provided by Invitrogen (Carlsbad, CA). The ELISA kit for murine TNF-α (70-EK282/3-96), IL-1β (70-EK201B/3-96), and IL-6 (70-EK206/3-96) were procured from Multi-Science Co. Ltd. (Hangzhou, China). Annexin V/PI staining kit (70-AP101-100) was bought from Multi-Science Co. Ltd. (Hangzhou, China). Antibody for Actin (sc-8432) was purchased from Santa Cruz (Santa Cruz, CA, USA). All other chemicals were supplied by Sigma-Aldrich (St. Louis, MO, USA).

### Cell Culture

Murine Raw 264.7 cells were obtained from the American Type Culture Collection (Rockville, MD). The cells were cultured in Dulbecco’s modified Eagle medium (DMEM) (Gibco, Grand Island, NY) containing 10% fetal bovine serum (FBS, Gibco, Grand Island, NY), 100 U/ml penicillin, and 100 mg/ml streptomycin in 5% CO2 at 37°C. Human monocytic THP-1 cell line was purchased from the American Type Culture Collection (Rockville, MD) and cultured in RPMI1640 (Gibco, Grand Island, NY) containing 10% fetal bovine serum (FBS, Gibco, Grand Island, NY), 100 U/ml penicillin, and 100 mg/ml streptomycin in 5% CO2 at 37°C. Bone marrow-derived macrophages (BMDMs) were prepared by minor changes based on previous work (Sutterwala et al., 1997). In brief, 21-ga needle was used to flush femurs using PBS. Medium comprising 10% FBS RPMI 1640 was prepared. The cells were cultured in the aforementioned medium at 37°C in 5% CO2 for 5 days. The adherent macrophages were washed twice with PBS, prior to culturing of the cells with fresh DMEM medium composing of 10% FBS and 10 ng/ml LPS and 10 ng/ml IFN-γ (M1-polarization) for 6 h.

### Quantitative Polymerase Chain Reaction (qPCR)

TRIzol (Invitrogen, Carlsbad, CA) was used to isolate total RNA from cells or tissues. Total RNA reverse was transcribed to complementary DNA (cDNA) and subjected to quantitative PCR *via* ABI 7500 detection system (Applied Biosystems, CA) using ChamQ Universal SYBR qPCR Master Mix (Vazyme, China). Bio-Rad CFX Manager Software was then applied to obtain threshold cycle numbers. The amplification program was 2 min at 95°C for 1 cycle followed by 10 s at 95°C for 40 cycle, 30 s at 60°C, and then 30 s at 72°C. Next, β-actin was used as endogenous control to normalize the entire sample’s difference in total RNA. Comparative CT method also referred to as 2^−ΔΔCT^ method, i.e., fold change = 2^−ΔΔCT^ = [(C_T_ gene of interest − C_T_ gene of actin control) sample A − (C_T_ gene of interest − C_T_ gene of actin control) sample B] was applied to calculate the relative gene expression. The primer sequences used in this study were as follows:

TNF-α, 5′-CGAGTGACAAGCCTGTAGCCC-3′ (forward) and 5′-GTCTTTGAGATCCATGCCGTTG-3′ (reverse);
IL-1β, 5′-CTTCAGGCAGGCAGTATCACTC-3′ (forward) and 5′-TGCAGTTGTCTAATGGGAACGT-3′ (reverse);
IL-6, 5′-ACAACCACGGCCTTCCCTAC-3′ (forward) and 5′-TCTCATTTCCACGATTTCCCAG-3′ (reverse);

### Cytokine Analysis by ELISA

The ELISA kits obtained from Multi-Science Co. Ltd. (Hangzhou, China) were used to quantify the mounts of TNF-α, IL-1β, and IL-6 in the supernatant or serum from mice. Specific experimental procedures were performed according to the manufacturer’s instructions.

### Western Blot

Samples were collected and lysed in a lysis buffer containing pro-tease inhibitor (protease inhibitor cocktail, Pierce). Afterwards, 10% sodium dodecyl sulfate-polyacrylamide gel electrophoresis (SDS-PAGE) was used to isolate the obtained protein lysate and then electrotransferred onto a polyvinylidene fluoride (PVDF) membrane (Millipore Corp., Bedford, MA). At room temperature, 5% skim milk was used to seal the membrane for 1 h. Subsequently, different antibodies were incubated with the secondary antibody after incubation at 4°C overnight, after which a suitable horseradish peroxidase (HRP)-conjugated secondary antibody was added. Protein bands were visualized using a Western blot detection system according to the specifications of the manufacturer (Cell Signaling Technology, MA).

### Immunofluorescent Assay

The paraformaldehyde (4%, PFA) was used to immobilize bone marrow-derived macrophages (BMDM) on coverslips, followed by permeation with 0.5% Triton X-100 for 20 min and was finally sealed with 3% bovine serum albumin (BSA) for 30 min. The cells were immunostained with anti-p65 Ab overnight. Then, Alexa Fluor 488-conjugated anti-rabbit IgG (Life technology, CA) was immunostained for 2 h. Afterwards, DAPI was applied to counterstain the coverslips and then imaged with a confocal laser scanning microscope (Olympus, Lake Success, NY).

### Flow Cytometry Analysis

Flow cytometry analysis was performed according to standard procedures. Briefly, cold PBS was used to harvest and wash cultured cells followed by staining with specific antibodies for 30 min at 4°C in the dark. Finally, the sample was analyzed *via* flow cytometry.

### Dextran Sulfate Sodium-Induced Colitis

The mouse model of acute colitis was induced *via* the addition of 3% DSS to the drinking water of the animals for 7 days. The C57BL/6 mice in the control group received the same drinking water without DSS (n=6 mice per group). Next, different doses of bilobalide (1.25, 2.5, 5 mg/kg) and 5-aminosalicylic acid (5-ASA) (100 mg/kg) were dissolved in the sodium salt of carboxymethyl cellulose (CMC-Na, 0.9%). Mice in the control and model groups were administered (i.g) once a day for 10 days. The bodyweight, the consistency of the stool and the presence of gross blood in feces and at the anus were observed daily. The disease activity index was calculated by assigning well-established and validated scores (Liu et al., 2013). The degree of diarrhea and blood in the stool as an evaluation index: a) degree of diarrhea (0 points = normal, 2 points = loose stools, 4 points = watery diarrhea); b) degree of hematochezia (0 points = no bleeding, 2 points = slight bleeding, 4 points = gross bleeding). On the 10^th^ day, the animals were sacrificed and the entire colon segment used for the *ex vivo* studies was quickly removed. They were then fixed in paraffin-embedded 10% normal buffered formalin, which was subsequently stained with hematoxylin and eosin and scored using an evaluated (blind) histology (0, no signs of inflammation; 1, low leukocyte infiltration; 2, moderate leukocyte infiltration; 3, high leukocyte infiltration, moderate fibrosis, high vascular density thickening of the colon wall, moderate goblet cell loss, and focal loss of crypts; as well as 4, transmural infiltrations, massive loss of goblet cell, extensive fibrosis, and diffuse loss of crypts).

### Immunohistochemical Analysis

In order to clarify the expression of p-p65, paraffin-embedded colon tissue sections (4–5 μm) were subjected to immunohistochemical analysis. The slides were dewaxed and rehydrated, after which they were sealed and incubated with p-p65 antibody at 4°C for a whole night. Next, streptavidin-HRP was used to incubate the slides for 40 min, then stained with 3, 3′ diaminobenzidine tetrahydrochloride (DAB) substrate (Santa Cruz Biotechnology, CA) and then counter-stained with hematoxylin. The images were taken with Olympus IX81.

### Statistical Analysis

All the data were presented as mean ± SEM of three independent experiments with each measurement performed in triplicate. One-way ANOVA analysis and student’s t-test were used for comparison between the control groups and the various experimental groups. Student’s t-test was used to compare between two groups. A P-value less than 0.05 was considered as statistically significant.

## Results

### Cytotoxicity of Bilobalide on Macrophages

**Figure 1:**
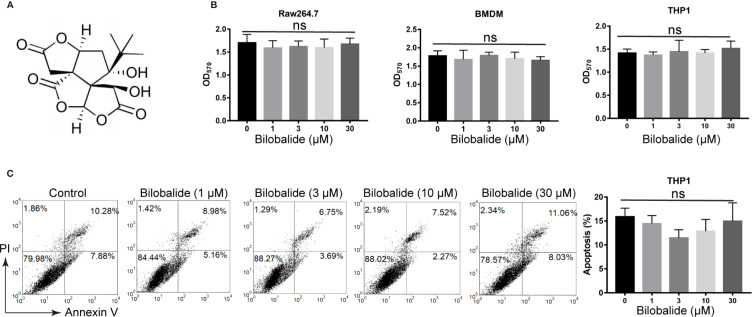
Bilobalide has no toxicity on macrophages. **(A)** Chemical structure of bilobalide. **(B)** Cell viability test of bilobalide on bone marrow-derived macrophages (BMDM), Raw 164.7, BMDM, and human monocytic leukemia cell line, THP1. The cells were incubated with various concentrations of bilobalide or the same volume of dimethyl sulfoxide (DMSO) and then tested for cell proliferation *via* 3-(4,5-dimethylthiazol-2-yl)-2,5-diphenyltetrazolium bromide (MTT) assay. **(C)** The cells were treated with 3, 10, and 30 μM bilobalide or the same volume of DMSO. Next, the cells were stained with annexin V/PI and assayed *via* flow cytometry. *P < 0.05 and **P < 0.01 compared with the control group.

The structure of bilobalide is shown in **Figure 1A**. It was observed that at concentration of 30 μM, the functional groups of bilobalide were nontoxic to Raw 264.7 cells (**Figure 1B**). Additionally, bilobalide did not inhibit the proliferation of both THP1 and BMDMs even at 30 μM (**Figure 1B**). Likewise, bilobalide did not induce marked apoptosis in BMDMs (**Figure 1C**). Collectively, these results indicated that bilobalide has no significant toxicity on macrophage.

### Effects of Bilobalide on Inflammatory Cytokine Expression and Differentiation of M1 Macrophage

**Figure 2:**
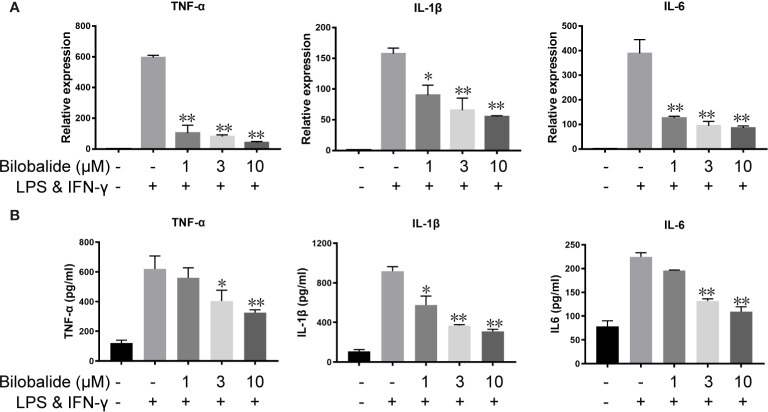
Bilobalide inhibited the cytokines expression of M1 macrophage. **(A)** Bone marrow-derived macrophages (BMDMs) were cultured with 1, 3, and 10 μM bilobalide in the absence or presence of 10 ng/ml lipopolysaccharide (LPS) and 10 ng/ml interferon-gamma (IFN)-γ (M1) for 6 h. The messenger RNA (mRNA) expression level of tumor necrosis factor (TNF)-α, interleukin (IL)-1β, and IL-6 were determined by quantitative PCR (QPCR). **(B)** BMDMs cells were treated with 3, 10, and 30 μM bilobalide in the presence of 10 ng/ml LPS and 10 ng/ml IFN-γ (M1) for 12 h. Cytokines level, IL-1β, IL-6, and TNF-α in the medium were assessed by ELISA. Data represented the mean ± SEM of three independent experiments in triplicate. *P < 0.05 and **P < 0.01 compared with LPS and IFN-γ group.

**Figure 3:**
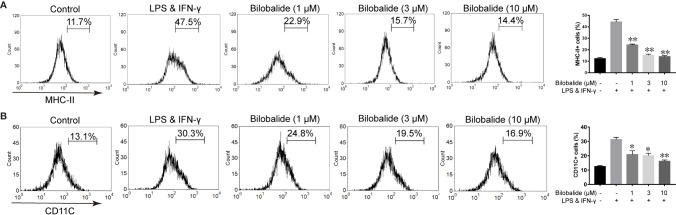
Bilobalide inhibited differentiation of M1 macrophage. **(A**, **B)** Bone marrow-derived macrophages (BMDMs) were treated with 1, 3, and 10 μM bilobalide in the presence of 10 ng/ml lipopolysaccharide (LPS) and 10 ng/ml interferon-gamma (IFN-γ) (M1) for 12 h. The expressions of major histocompatibility complex II (MHC-II) and CD11c were analyzed through flow cytometry. Data was summarized as a histogram of mean ± SEM of three independent experiments. *P < 0.05 and **P < 0.01 compared with LPS and IFN-γ group.

Based on the aforementioned method and the results obtained, the macrophage polarization model in BMDMs cells was established to further evaluate the function of bilobalide. As shown in **Figure 2**, messenger RNA (mRNA) and protein expression of IL-1β, IL-6, and TNF-α in M1-polarized BMDMs cells were inhibited by bilobalide in a dose-dependent manner. Further, bilobalide treatment dose-dependently decreased the positive cells of MHC-II and CD11c (M1-macrophages markers) (**Figure 3**). Overall, these results demonstrated the anti-inflammatory effect of bilobalide was due to the inhibition of differentiation of M1-polarized macrophages.

### Bilobalide Hampered Differentiation of M1 Macrophage *via* Inhibiting NF-κB Signaling

**Figure 4:**
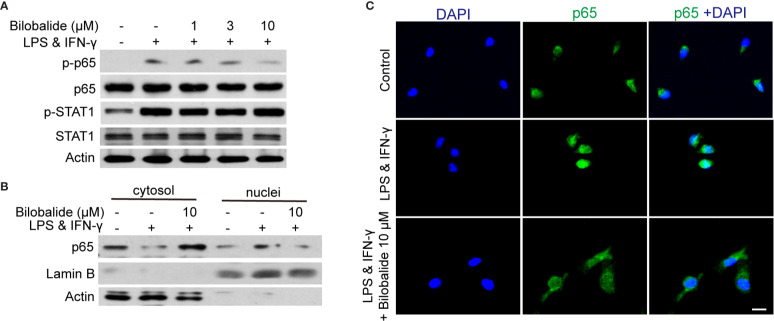
Bilobalide inhibited NF-κB signaling which hampered differentiation of M1 macrophage. **(A)** Bone marrow-derived macrophages (BMDMs) were treated with 1, 3, and 10 μM bilobalide in the presence of 10 ng/ml lipopolysaccharide (LPS) and 10 ng/ml interferon-gamma (IFN-γ) (M1) for 30 min. The expression of MyD88, TRAF6, p-STAT1, STAT1, p-p65, and p65 were determined by western blot. **(B)** BMDMs were treated with 10 μM bilobalide in the presence of 10 ng/ml LPS and 10 ng/ml IFN-γ (M1) for 30 min. The cytosol and nuclei fraction were isolated and the level of p65 was determined by western blot. **(C)** The BMDMs were cultured as described above and stained with p65. The nuclei were stained with 4′, 6-diamidino-2-phenylindole (DAPI) (blue). Scale bar: 10 μm.

The canonical NF-κB pathway has been recognized as a crucial transcription factor in regulating macrophage polarization (Kuhnemuth et al., 2015). In the following experiments, STAT1 and NF-κB signaling pathways were examined. The result indicated that p65 phosphorylation was significantly inhibited, whereas the phosphorylated STAT1 was not altered by bilobalide treatment (**Figure 4A**). In order to further assess the activation of p65, the location of p65 was examined by cellular components isolation and immunofluorescence. As shown in **Figure 4B**, under normal condition, most of p65 was located in the cytosol. After stimulation, p65 translocation into nucleus was markedly inhibited by bilobalide treatment. Besides, the immunofluorescence analysis also confirmed that the bilobalide treatment could obviously inhibit the accumulation of NF-κB p65 in the nuclei of BMDMs (**Figure 4C**). Altogether, these results verified that bilobalide could inhibit the NF-κB signaling and thereby hampered differentiation of M1 macrophage.

### Bilobalide Ameliorated Dextran Sulfate Sodium-Mediated Colitis in Mice

**Figure 5:**
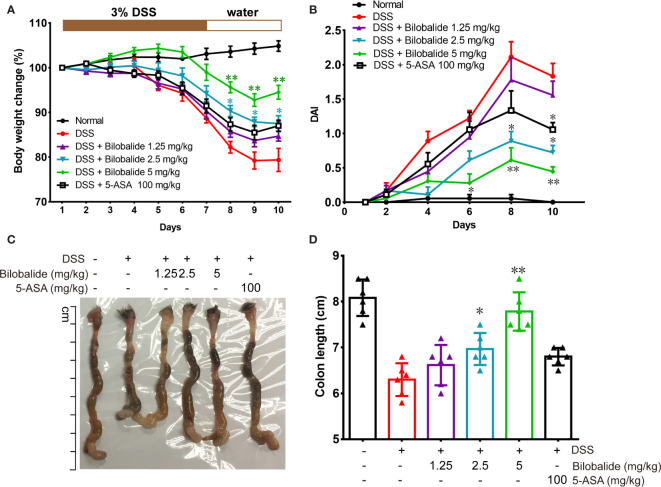
Bilobalide alleviated dextran sulfate sodium (DSS)-induced experimental colitis. **(A)** The body weight of mice was measured and presented as a percentage of the original body weight. **(B)** The calculated disease activity index. **(C)** Photograph of colon. **(D)** Length of colon when the mouse is sacrificed. Values were presented as mean ± SEM of 6 mice/group. *P < 0.05, **P < 0.01 compared with DSS-treated group.

The therapeutic potential of bilobalide on acute colitis was then detected in DSS-induced experimental colitis model. It was observed that the body weight of the mice substantially decreased after DSS administration compared with the normal group (drinking water) (**Figure 5A**). However, compared with the DSS group, body weights of the mice were improved significantly when they were treated with bilobalide (**Figure 5A**). Additionally, cumulative DAI, which is usually applied as surrogate of degree of disease development, reduced markedly in DDS + bilobalide group compared with DSS group (**Figure 5B**). Notably, the administration of DSS resulted in remarkable decrease in the length of the colon, whereas bilobalide treatment significantly improved the contractible of the colon (**Figures 5C, D**). However, the 5-aminosalicylic acid (5-ASA) had no obvious influence on the colon length in comparison with the DSS challenged mice. Collectively, these results demonstrate that bilobalide could improve the severity of colitis in DSS-induced colitic mice, which may probably be attributed to the anti-inflammatory properties of bilobalide.

### Effect of Bilobalide on Histological Alteration

**Figure 6:**
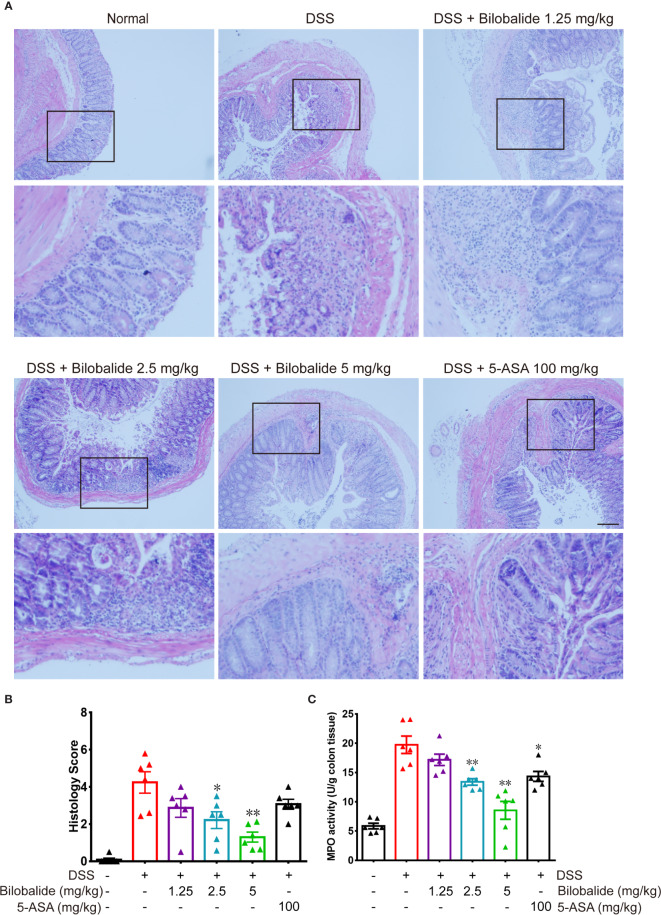
Bilobalide alleviated dextran sulfate sodium (DSS)-induced colon damage in mice. **(A**, **B)** Light microscopy assessment of paraffin section of colon tissue after H & E staining. Scale bar: 50 μm. **(C)** The myeloperoxidase (MPO) activity in colonic tissue was examined. Values were mean ± SEM of 6 mice/group. *P < 0.05, **P < 0.01 compared with DSS-treated group.

In order to further confirm the colitis improvement achieved by bilobalide, histological results are presented in **Figure 6**. According to the degree of inflammatory cells infiltration, the colon inflammation was divided into five levels. There was no inflammatory cells in the normal mucosa, submucosal, muscularis propria, and serosa in normal group. Instead, multiple large ulcers with transmural inflammation, necrosis of the entire wall or part of it, massive depletion of crypt, goblet cells, and infiltration of inflammatory cells, e.g., neutrophils and eosinophils were observed in DSS challenged mice. The pathological changes with the administration of bilobalide demonstrated relatively low histological injury score compared with the DSS group (**Figures 6A, B**). The levels of myeloperoxidase (MPO) activity in the colon tissue of DSS-induced mice are shown in **Figure 6C**. The increased activity of MPO could mainly be attributed to the DSS administration in the colon tissue compared with the normal group. MPO, as a kind of hemeprotein is mainly present in eosinophilic granules of neutrophils and is used as an indirect measure of the intensity of tissue inflammation. As shown in **Figure 6C**, the MPO activity was obviously decreased at 2.5 and 5 mg/kg group in comparison with the DSS group. The 5-ASA treated group had a relatively poorly inhibitory effect on MPO positive cells compared with the DSS group. Overall, these findings showed that bilobalide could effectively relieve symptoms of colitis effectively in mice like bloody diarrhea and MPO expression in the acute phase of colitis in colonic tissue.

### Effect of Bilobalide on Cytokine Levels and NF-κB Activation

**Figure 7:**
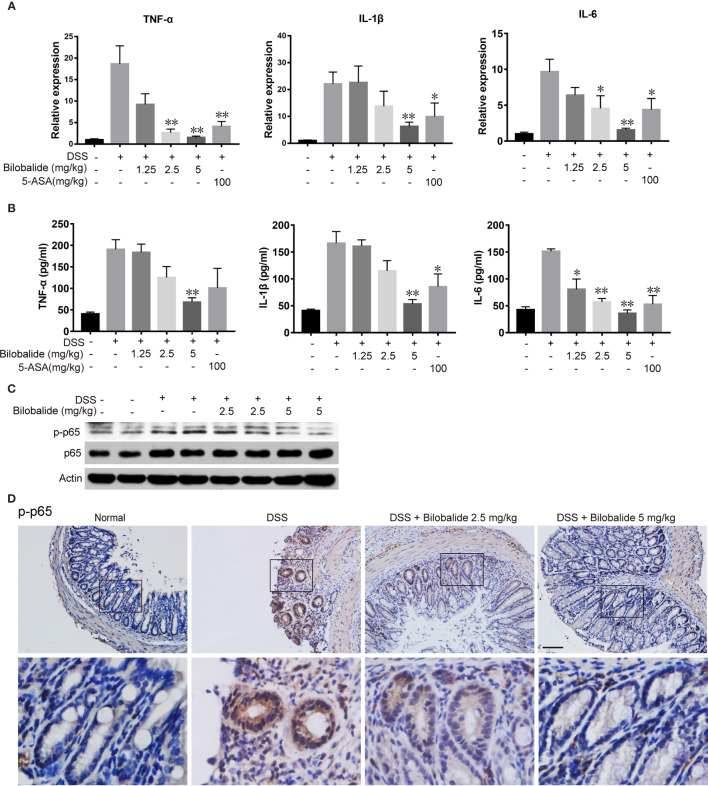
Bilobalide inhibited M1 macrophage differentiation in dextran sulfate sodium (DSS)-induced colitis in mice. **(A)** Quantitative determination of mRNA expression of tumor necrosis factor (TNF)-α, interleukin (IL)-1β, and IL-6 in colon tissue. **(B)** TNF-α, IL-1β, and IL-6 in serum were measured by ELISA. Values were presented as mean ± SEM of 6 mice/group. *P < 0.05, **P < 0.01 compared with DSS-treated group. **(C**, **D)** The p-p65 expression in colon tissue were examined by western blotting and immunohistochemistry (IHC). Scale bar: 50 μm.

To examine whether the improvements in colitis were associated with decreased inflammation, we subsequently analyzed the expression of cytokines. As indicated in **Figure 7A**, the mRNA expression of pro-inflammatory cytokines (TNF-α, IL-1β, and IL-6) in DSS mice increased significantly compared with the normal mice. The changes in cytokine levels were obviously diminished compared with the DSS control group when bilobalide (5 mg/kg) was administered. Next, ELISA was applied to evaluate the serum levels of pro-inflammatory cytokines including TNF-α, IL-6, and IL-1β. As depicted in **Figure 7B**, these cytokines were increased in DSS group comparable to the normal group. Meanwhile, treatment with high dose bilobalide demonstrated a substantial reduction in the levels of TNF-α, IL-6, and IL-1β compared with the DSS group. More importantly, it was observed that the cytokines were declined slightly n 5-ASA group. Likewise, the lower dose of bilobalide (1.25 mg/kg) had no obvious influence on the levels of cytokines. Also, western blot assay showed that bilobalide also decreased the phosphorylation of p65 in the colons of DSS-induced group (**Figures 7C, D**), coincidently with the data *in vitro*. Thus, this work strongly indicates that the suppression of NF-κB signaling activation may induce the anti-ulcerative colitis effect of bilobalide. Altogether, these results demonstrated that bilobalide may alleviate DSS-induced colitis. Therefore, the relationship between the protective effect of bilobalide in colitic mice and its inhibition in M1-induced inflammation was in agreement.

## Discussion

Pathologically, IBD is a chronic inflammatory disease of the gastrointestinal tract. It is a well-established risk factor of colorectal cancer and has a high prevalence worldwide. Macrophages have an important role in the pathogenesis of IBD. Natural products have drawn lots of attentions for their possible therapeutic activities. Bilobalide is a sesquiterpenoid lactone from *G. biloba.* Herein, the anti-colitic activity and possible mechanism was examined in this study.

Firstly, we evaluated the *in vivo* anti-inflammatory activity of bilobalide. Bilobalide decreased both them RNA and protein levels of pro-inflammatory factors in concentration dependent manner. To confirm the underlying mechanism of bilobalide, we measured the phosphorylation levels of two transcription factors (STAT1 and p65). The results suggested that bilobalide exerted a more significant inhibition on p65, relative to that of STAT1. Based on this result, we therefore propose that bilobalide mainly modulated the p65 signaling pathway rather than that of the STAT1 pathway.

Based on the results from the *in vitro* experiments using (cell line model), we further comprehensively evaluated the *in vivo* anti-inflammatory activity of bilobalide in DSS-induced colitis model. Bilobalide significantly ameliorated the DSS-induced loss of bodyweight, colon shortening, and colonic inflammation. The pro-inflammatory cytokines such as TNF-α, IL-6, and IL-1β have been shown to exhibit potential efficacy against the pathogenesis of IBD (Nenci et al., 2007). Available literatures has also confirmed that pro-inflammatory cytokines interaction with the intestinal mucosal immune system at the intestinal interface may culminate in the disruption of tight junction proteins and impact intestinal homeostasis (Abraham and Medzhitov, 2011). Meanwhile, the characteristics of colitis coupled with many other chronic inflammatory diseases are characterized with the production of more pro-inflammatory cytokines such as IL-6, IL-1β, and TNF-α in serum and tissue (Muzes et al., 2012). Also, bilobalide significantly reduced M1-type mRNA expression.

The polarization of macrophages which is responsible for the activation of macrophages activate a range of signal pathways to elicit changes in gene expression coupled with promotion of the corresponding functional properties. This phenomenon usually occurs when the microenvironment alters, and it is coordinated by a large range of mediators and regulators (Sica and Mantovani, 2012; Mantovani et al., 2013) but stimulated by different inducers. Microbial components like LPS, interact with toll-like receptor 4 (TLR4) to skew macrophages to the proinflammatory phenotype *via* the NF-κB signal pathway (Lawrence and Natoli, 2011). The NF-κB modulates a diverse spectrum of biological processes and operates as a central regulator of inflammation (Tian et al., 2005; Wang et al., 2017). The NF-κB signaling cascade has been well studied in IBD treatment and is often dysregulated in patients, resulting in aberrant cytokine and chemokine production in the gut (Zaidi and Wine, 2018; Benary and Wolf, 2019). Inflammation and carcinogenesis have been associated with NF-κB signaling (Huang et al., 2014). The activation of the NF-κB signaling pathway triggers an important inflammatory response, which is closely related to colorectal tumorigenesis (Ye et al., 2019). Existing literature has reviewed that the anti-inflammatory effects of some drugs against DSS-induced colitis models are *via* the inhibition of NF-κB signaling pathway (Cheon et al., 2006; Zhou et al., 2017; Zhu et al., 2019). The activation of NF-κB signal pathway was suppressed by bilobalide based on the studied mechanisms. However, the exact proteins targeted by bilobalide still need further investigation.

In summary, bilobalide has been proved to be a low cellulotoxic and strong anti-inflammatory agent. Its ability to restraining M1 macrophage polarization by hampering the NF-κB pathways was manifested *in vitro* and *in vivo*, suggesting a potential use of bilobalide as therapeutic option for in anti-inflammation therapy.

## Data Availability Statement

The datasets generated for this study are available on request to the corresponding author.

## Ethics Statement

The animal study was reviewed and approved by The animal experiments were approved by Animal Experimental Ethical Committee of Southeast University and carried out at Laboratory Animal Research Centre of Jiangsu University (NO. 2018–0553).

## Author Contributions

HZ consulted the literature and wrote experimental protocols, purchased reagent supplies and laboratory animals, participated in the experiments process and wrote the manuscript. NC consulted references, bought reagent supplies and laboratory animals, participated in the experiments process and wrote the manuscript. ZY, XF, XY, HL, and ZH participated in the experiments process, did optimization of experimental scheme and wrote the manuscript. ZJ provided guidance and assistance in the experimental process and help in the writing of the article. All the authors read and approved the final manuscript.

## Funding

This work was supported by Medical clinical science and technology development fund of Jiangsu University (JLY20180033).

## Conflict of Interest

The authors declare that the research was conducted in the absence of any commercial or financial relationships that could be construed as a potential conflict of interest.

## References

[B1] Abdel-Salam O. M. E. Baiuomy A. R. El-batran S. Arbid M. S. (2004) Evaluation of the anti-inflammatory, anti-nociceptive and gastric effects of Ginkgo biloba in the rat. Pharmacol. Res. 49 (2), 133–142. 10.1016/j.phrs.2003.08.004 14643693

[B2] Abraham C. Medzhitov R. (2011). Interactions between the host innate immune system and microbes in inflammatory bowel disease. Gastroenterology 140 (6), 1729–1737. 10.1053/j.gastro.2011.02.012 21530739 PMC4007055

[B3] Al-Adwani D. G. Renno W. M. Orabi K. Y. (2019). Neurotherapeutic effects of Ginkgo biloba extract and its terpene trilactone, ginkgolide B, on sciatic crush injury model: A new evidence. PloS One 14 (12), e022662. 10.1371/journal.pone.0226626 PMC6932810 31877172

[B4] Barger S. W. Horster D. Furukawa K. Goodman Y. Krieglstein J. Mattson M. P. (1995). Tumor necrosis factors alpha and beta protect neurons against amyloid beta-peptide toxicity: evidence for involvement of a kappa B-binding factor and attenuation of peroxide and Ca2+ accumulation. Proc. Natl. Acad. Sci. U. S. A. 92 (20), 9328–9332. 10.1073/pnas.92.20.9328 7568127 PMC40978

[B5] Benary U. Wolf J. (2019). Controlling Nuclear NF-kappaB Dynamics by beta-TrCP-Insights from a Computational Model. Biomedicines 7 (2), 40. 10.3390/biomedicines7020040 PMC6631534 31137887

[B6] Chassaing B. Aitken J. D. Malleshappa M. Vijay-Kumar M. (2014). Dextran Sulfate Sodium (DSS)-Induced Colitis in Mice. In. Curr. Protoc. Immunol. 104, 15.25.11–15.25.14. 10.1002/0471142735.im1525s104 PMC3980572 24510619

[B7] Cheon J. H. Kim J. S. Kim J. M. Kim N. Jung H. C. Song I. S. (2006). Plant sterol guggulsterone inhibits nuclear factor-kappaB signaling in intestinal epithelial cells by blocking IkappaB kinase and ameliorates acute murine colitis. Inflammation Bowel Dis. 12 (12), 1152–1161. 10.1097/01.mib.0000235830.94057.c6 17119390

[B8] Cheung F. Siow Y. L. Wei Z. C. (1999). Inhibitory effect of Ginkgo biloba extract on the expression of inducible nitric oxide synthase in endothelial cells. Biochem. Pharmacol. 58 (10), 1665–1673. 10.1016/S0006-2952(99)00255-5 10535759

[B9] Di Martino L. Dave M. Menghini P. Xin W. Arseneau K. O. Pizarro T. T. (2016). Protective Role for TWEAK/Fn14 in Regulating Acute Intestinal Inflammation and Colitis-Associated Tumorigenesis. Cancer Res. 76 (22), 6533–6542. 10.1158/0008-5472.CAN-16-0400 27634763 PMC5290134

[B10] Feng Y. He X. Luo S. Chen X. Long S. Liang F. (2019). Chronic colitis induces meninges traffic of gut-derived T cells, unbalances M1 and M2 microglia/macrophage and increases ischemic brain injury in mice. Brain Res. 1707, 8–17. 10.1016/j.brainres.2018.11.019 30445026

[B11] Goldie M. Dolan S. (2013). Bilobalide, a unique constituent of Ginkgo biloba, inhibits inflammatory pain in rats. Behav. Pharmacol. 24 (4), 298–306. 10.1097/FBP.0b013e32836360ab 23838965

[B12] Haffner-Luntzer M. Foertsch S. Fischer V. Prystaz K. Tschaffon M. Modinger Y. (2019). Chronic psychosocial stress compromises the immune response and endochondral ossification during bone fracture healing via beta-AR signaling. Proc. Natl. Acad. Sci. U. S. A. 116 (17), 8615–8622. 10.1073/pnas.1819218116 30948630 PMC6486758

[B13] Huang H.-Y. Zhang Z.-J. Cao C.-B. Wang N. Liu F.-F. Peng J.-Q. (2014). The TLR4:NF-κB signaling pathway mediates the growth of colon cancer. Eur. Rev. Med. Pharmacol. Sci. 18 (24), 3834–3843. 25555874

[B14] Ilieva I. Ohgami K. Shiratori K. Koyama Y. Yoshida K. Kase S. (2004) The effects of Ginkgo biloba extract on lipopolysaccharide-induced inflammation in vitro and in vivo. Exp. Eye Res. 79 (2), 0–187. 10.1016/j.exer.2004.03.009 15325565

[B15] Keane T. J. Dziki J. Castelton A. Faulk D. M. Messerschmidt V. Londono R. (2017). Preparation and characterization of a biologic scaffold and hydrogel derived from colonic mucosa. J. BioMed. Mater. Res. B Appl. Biomater. 105 (2), 291–306. 10.1002/jbm.b.33556 26506408

[B16] Kim T. H. Kang M. S. Mandakhbayar N. El-Fiqi A. Kim H. W. (2019). Anti-inflammatory actions of folate-functionalized bioactive ion-releasing nanoparticles imply drug-free nanotherapy of inflamed tissues. Biomaterials 207, 23–38. 10.1016/j.biomaterials.2019.03.034 30952042

[B17] Kobuchi H. Droy-Lefaix M. T. Christen Y. Packer L. (1997). Ginkgo biloba extract (EGb 761): inhibitory effect on nitric oxide production in the macrophage cell line RAW 264.7. Biochem. Pharmacol. 53 (6), 897–903. 10.1016/S0006-2952(96)00873-8 9113109

[B18] Kuhnemuth B. Muhlberg L. Schipper M. Griesmann H. Neesse A. Milosevic N. (2015). CUX1 modulates polarization of tumor-associated macrophages by antagonizing NF-kappaB signaling. Oncogene 34 (2), 177–187. 10.1038/onc.2013.530 24336331

[B19] Lawrence T. Natoli G. (2011). Transcriptional regulation of macrophage polarization: enabling diversity with identity. Nat. Rev. Immunol. 11 (11), 750–761. 10.1038/nri3088 22025054

[B20] Li X. Huang L. Liu G. Fan W. Li B. Liu R. (2020). Ginkgo diterpene lactones inhibit cerebral ischemia/reperfusion induced inflammatory response in astrocytes via TLR4/NF-kappaB pathway in rats. J. Ethnopharmacol. 249, 112365. 10.1016/j.jep.2019.112365 31678414

[B21] Lin Y. Yang X. Yue W. Xu X. Li B. Zou L. (2014). Chemerin aggravates DSS-induced colitis by suppressing M2 macrophage polarization. Cell Mol. Immunol. 11 (4), 355–366. 10.1038/cmi.2014.15 24727542 PMC4085517

[B22] Liu W. Guo W. Wu J. Luo Q. Tao F. Gu Y. (2013). *A* novel benzo[d]imidazole derivate prevents the development of dextran sulfate sodium-induced murine experimental colitis via inhibition of NLRP3 inflammasome. Biochem. Pharmacol. 85 (10), 1504–1512. 10.1016/j.bcp.2013.03.008 23506741

[B23] Lokhonina A. Elchaninov A. Fatkhudinov T. Makarov A. Arutyunyan I. Grinberg M. (2019). Activated Macrophages of Monocytic Origin Predominantly Express Proinflammatory Cytokine Genes, Whereas Kupffer Cells Predominantly Express Anti-Inflammatory Cytokine Genes. BioMed. Res. Int. 2019, 3912142. 10.1155/2019/3912142 30949499 PMC6425426

[B24] Mantovani A. Biswas S. K. Galdiero M. R. Sica A. Locati M. (2013). Macrophage plasticity and polarization in tissue repair and remodelling. J. Pathol. 229 (2), 176–185. 10.1002/path.4133 23096265

[B25] Medzhitov R. (2008). Origin and physiological roles of inflammation. Nature 454 (7203), 428–435. 10.1038/nature07201 18650913

[B26] Mei Y. Fang C. Ding S. Liu X. Hu J. Xu J. (2019). PAP-1 ameliorates DSS-induced colitis with involvement of NLRP3 inflammasome pathway. Int. Immunopharmacol. 75, 105776. 10.1016/j.intimp.2019.105776 31351364

[B27] Middleton E. (1992). Effects of flavonoids on immune and inflammatory cell functions. Biochem. Pharmacol. 43 (6), 1167–1179. 10.1016/0006-2952(92)90489-6 1562270

[B28] Muzes G. Molnar B. Tulassay Z. Sipos F. (2012). Changes of the cytokine profile in inflammatory bowel diseases. World J. Gastroenterol. 18 (41), 5848–5861. 10.3748/wjg.v18.i41.5848 23139600 PMC3491591

[B29] Nenci A. Becker C. Wullaert A. Gareus R. van Loo G. Danese S. (2007). Epithelial NEMO links innate immunity to chronic intestinal inflammation. Nature 446 (7135), 557–561. 10.1038/nature05698 17361131

[B30] Nunes N. S. Chandran P. Sundby M. Visioli F. da Costa Goncalves F. Burks S. R. (2019). Therapeutic ultrasound attenuates DSS-induced colitis through the cholinergic anti-inflammatory pathway. EBioMedicine 45, 495–510. 10.1016/j.ebiom.2019.06.033 31253515 PMC6642284

[B31] Okayasu I H. S. Yamada M. Ohkusa T. Inagaki Y. Nakaya R. (1990). A Novel Method in the Induction of Reliable Experimental Acute and Chronic Ulcerative Colitis in Mice. Gastroenterology 98 (3), 694–702. 10.1016/0016-5085(90)90290-H 1688816

[B32] Ong Lai Teik D. Lee X. S. Lim C. J. Low C. M. Muslima M. Aquili L. (2016). Ginseng and Ginkgo Biloba Effects on Cognition as Modulated by Cardiovascular Reactivity: A Randomised Trial. PloS One 11 (3), e0150447. 10.1371/journal.pone.0150447 26938637 PMC4777384

[B33] Piazza S. Pacchetti B. Fumagalli M. Bonacina F. Dell’Agli M. Sangiovanni E. (2019). Comparison of Two Ginkgo biloba L. Extracts on Oxidative Stress and Inflammation Markers in Human Endothelial Cells. Mediators Inflamm. 2019, 6173893. 10.1155/2019/6173893 31341420 PMC6614955

[B34] Shi J. Zhang X. Jiang L. Zhang L. Dong Y. Midgley A. C. (2019). Regulation of the inflammatory response by vascular grafts modified with Aspirin-Triggered Resolvin D1 promotes blood vessel regeneration. Acta Biomater. 97, 360–373. 10.1016/j.actbio.2019.07.037 31351251

[B35] Sica A. Mantovani A. (2012). Macrophage plasticity and polarization: in vivo veritas. J. Clin. Investig. 122 (3), 787–795. 10.1172/jci59643 22378047 PMC3287223

[B36] Song S. An J. Li Y. Liu S. (2019). Electroacupuncture at ST-36 ameliorates DSS-induced acute colitis via regulating macrophage polarization induced by suppressing NLRP3/IL-1beta and promoting Nrf2/HO-1. Mol. Immunol. 106, 143–152. 10.1016/j.molimm.2018.12.023 30610999

[B37] Sun X. Stefanetti G. Berti F. Kasper D. L. (2019). Polysaccharide structure dictates mechanism of adaptive immune response to glycoconjugate vaccines. Proc. Natl. Acad. Sci. U. S. A. 116 (1), 193–198. 10.1073/pnas.1816401115 30510007 PMC6320544

[B38] Sutterwala F. S. Noel G. J. Clynes R. Mosser D. M. (1997). Selective suppression of interleukin-12 induction after macrophage receptor ligation. J. Exp. Med. 185 (11), 1977–1985. 10.1084/jem.185.11.1977 9166427 PMC2196339

[B39] Tian B. Nowak D. E. Jamaluddin M. Wang S. Brasier A. R. (2005). Identification of direct genomic targets downstream of the nuclear factor-kappaB transcription factor mediating tumor necrosis factor signaling. J. Biol. Chem. 280 (17), 17435–17448. 10.1074/jbc.M500437200 15722553

[B40] Tonussi C. R. Ferreira S. H. (1992) Rat knee-joint carrageenin incapacitation test: an objective screen for central and peripheral analgesics. Pain 48 (3), 421–427. 10.1016/0304-3959(92)90095-S 1594266

[B41] Wadsworth T. L. (2001). Effects of ginkgo biloba extract (EGb 761) and quercetin on lipopolysaccharide-induced signaling pathways involved in the release of tumor necrosis factor-alfa. Biochem. Pharmacol. 62 (7), 963–974. 10.1016/S0006-2952(01)00734-1 11543732

[B42] Wang L. Zhai D. S. Ruan B. J. Xu C. M. Ye Z. C. Lu H. Y. (2017). Quaking Deficiency Amplifies Inflammation in Experimental Endotoxemia via the Aryl Hydrocarbon Receptor/Signal Transducer and Activator of Transcription 1-NF-kappaB Pathway. Front. Immunol. 8, 1754. 10.3389/fimmu.2017.01754 29276519 PMC5727050

[B43] Wang S. W. Bai Y. F. Weng Y. Y. Fan X. Y. Huang H. Zheng F. (2019). Cinobufacini Ameliorates Dextran Sulfate Sodium-Induced Colitis in Mice through Inhibiting M1 Macrophage Polarization. J. Pharmacol. Exp. Ther. 368 (3), 391–400. 10.1124/jpet.118.254516 30606760

[B44] Wei Z. Peng Q. Lau B. H. Shah V. (1999). Ginkgo biloba inhibits hydrogen peroxide-induced activation of nuclear factor kappa B in vascular endothelial cells. Gen. Pharmacol. 33 (5), 369–375. 10.1016/s0306-3623(99)00027-0 10553877

[B45] Wu Y. Z. Li S. Q. Zu X. G. Du J. Wang F. F. (2008). Ginkgo biloba extract improves coronary artery circulation in patients with coronary artery disease: contribution of plasma nitric oxide and endothelin-1. Phytother. Res. PTR 22 (6), 734–739. 10.1002/ptr.2335 18446847

[B46] Ye X. Wu H. Sheng L. Liu Y. X. Ye F. Wang M. (2019). Oncogenic potential of truncated RXRalpha during colitis-associated colorectal tumorigenesis by promoting IL-6-STAT3 signaling. Nat. Commun. 10 (1), 1463. 10.1038/s41467-019-09375-8 30931933 PMC6443775

[B47] Yu T. Yu H. Zhang B. Wang D. Li B. Zhu J. (2019). Promising Neuroprotective Function for M2 Microglia in Kainic Acid-Induced Neurotoxicity Via the Down-Regulation of NF-kappaB and Caspase 3 Signaling Pathways. Neuroscience 406, 86–96. 10.1016/j.neuroscience.2019.03.002 30858108

[B48] Zaidi D. Wine E. (2018). Regulation of Nuclear Factor Kappa-Light-Chain-Enhancer of Activated B Cells (NF-kappabeta) in Inflammatory Bowel Diseases. Front. Pediatr. 6, 317. 10.3389/fped.2018.00317 30425977 PMC6218406

[B49] Zeng X. P. Wang L. J. Guo H. L. He L. Bi Y. W. Xu Z. L. (2019). Dasatinib Ameliorates Chronic Pancreatitis Induced by Caerulein via Anti-fibrotic and Anti-inflammatory Mechanism. Pharmacol. Res. 147, 104357. 10.1016/j.phrs.2019.104357 31356863

[B50] Zhang H. F. Huang L. B. Zhong Y. B. Zhou Q. H. Wang H. L. Zheng G. Q. (2016). An Overview of Systematic Reviews of Ginkgo biloba Extracts for Mild Cognitive Impairment and Dementia. Front. Aging Neurosci. 8, 276. 10.3389/fnagi.2016.00276 27999539 PMC5138224

[B51] Zhang B. Wei Y. Z. Wang G. Q. Li D. D. Shi J. S. Zhang F. (2018). Targeting MAPK Pathways by Naringenin Modulates Microglia M1/M2 Polarization in Lipopolysaccharide-Stimulated Cultures. Front. Cell Neurosci. 12, 531. 10.3389/fncel.2018.00531 30687017 PMC6336899

[B52] Zhang L. Liu J. Ge Y. Liu M. (2019). Ginkgo biloba Extract Reduces Hippocampus Inflammatory Responses, Improves Cardiac Functions And Depressive Behaviors In A Heart Failure Mouse Model. Neuropsychiatr. Dis. Treat. 15, 3041–3050. 10.2147/ndt.S229296 31754303 PMC6825506

[B53] Zhou J. Tan L. Xie J. Lai Z. Huang Y. Qu C. (2017). Characterization of brusatol self-microemulsifying drug delivery system and its therapeutic effect against dextran sodium sulfate-induced ulcerative colitis in mice. Drug Deliv. 24 (1), 1667–1679. 10.1080/10717544.2017.1384521 29078713 PMC8253134

[B54] Zhu Y. Li X. Chen J. Chen T. Shi Z. Lei M. (2016). The pentacyclic triterpene Lupeol switches M1 macrophages to M2 and ameliorates experimental inflammatory bowel disease. Int. Immunopharmacol. 30, 74–84. 10.1016/j.intimp.2015.11.031 26655877

[B55] Zhu Y. Zhou J. Feng Y. Chen L. Zhang L. Yang F. (2018). Control of Intestinal Inflammation, Colitis-Associated Tumorigenesis, and Macrophage Polarization by Fibrinogen-Like Protein 2. Front. Immunol. 9, 87. 10.3389/fimmu.2018.00087 29441068 PMC5797584

[B56] Zhu L. Gu P. Shen H. (2019). Protective effects of berberine hydrochloride on DSS-induced ulcerative colitis in rats. Int. Immunopharmacol. 68, 242–251. 10.1016/j.intimp.2018.12.036 30743078

[B57] Zigron S. Bronstein J. (2019). “Help is where you find it”: The role of weak ties networks as sources of information and support in virtual health communities. J. Assoc. Inf. Sci. Technol. 70 (2), 130–139. 10.1002/asi.24106

